# The Current Role of Contrast-Enhanced Ultrasound (CEUS) in the Diagnosis and Staging of Bladder Cancer: A Review of the Available Literature

**DOI:** 10.3390/life14070857

**Published:** 2024-07-09

**Authors:** Valerio Santarelli, Davide Rosati, Vittorio Canale, Stefano Salciccia, Giovanni Di Lascio, Giulio Bevilacqua, Antonio Tufano, Alessandro Sciarra, Vito Cantisani, Giorgio Franco, Martina Moriconi, Giovanni Battista Di Pierro

**Affiliations:** 1 Department of Maternal-Infant and Urological Sciences, “Sapienza” Rome University, Policlinico Umberto I Hospital, 00185 Rome, Italy; 2 Department of Radiology, Oncology and Pathology, University La Sapienza of Rome, 00185 Roma, Italy

**Keywords:** CEUS, ultrasound, bladder cancer, review, diagnosis, staging

## Abstract

Contrast-enhanced ultrasound (CEUS) is an advanced imaging technique that integrates conventional US with the intravenous injection of specific US contrast agents (UCAs), combining the non-invasiveness of US with the higher accuracy of contrast-enhanced imaging. In contrast with magnetic resonance imaging (MRI), computed tomography (CT) and cystoscopy, CEUS has few contraindications, and UCAs are non-nephrotoxic agents that can be safely used in patients with kidney failure. CEUS is a well-established method for the detection of liver lesions and for echocardiography, and its indications are expanding. The updated 2018 WFUMB-EFSUMB guidelines have added the urinary bladder under non-hepatic applications of CEUS. The technique is able to distinguish between benign tissue, such as clots or hematoma, and malignant lesions by perfusing the mass with contrast agent. Thanks to the different perfusion rates of the various layers of the bladder wall, CEUS is also able to predict tumor invasion depth and stage. Despite that, current urological guidelines do not include CEUS as a plausible imaging technique for bladder urothelial carcinoma. The main reason for this omission might be the presence of scarce randomized evidence and the absence of large validated series. In this review, we describe the rationale behind the use of CEUS in bladder cancer and the added value of this imaging technique in the detection and staging of bladder lesions. In addition, we researched the available literature on the topic and then described the results of randomized clinical trials and a meta-analysis investigating the accuracy of CEUS in bladder cancer diagnosis and staging. The reported studies show that CEUS is a highly accurate diagnostic and staging tool for BC, reaching levels of specificity and sensitivity in differentiating between Ta-T1, or low-grade BC, and T2, or high-grade BC, that are comparable to those shown by the reference standard methods. Nonetheless, several limitations were found and are highlighted in this review. The aim of this study is to further validate and promote the use of CEUS as a quick, economic and effective diagnostic tool for this high-impact disease.

## 1. Introduction

Bladder cancer (BC) is the tenth most common cancer worldwide [1]. Men have a higher incidence of disease, whereas women exhibit a higher disease-specific mortality [2]. Smoking and occupational exposure are the main risk factors, which explain the differences in incidence among genders and regions, together with access to healthcare [3]. Urothelial carcinoma represents more than 90% of bladder cancers and can be classified as either high-grade BC (WHO grade II and III) or low-grade BC (WHO grade I), with significant differences in terms of recurrence and progression rates [4]. The Tumor, Node, Metastasis (TNM) classification (2017, 8th edition) is the most widely accepted method for BC staging [5]. Tumors confined to the mucosa or the lamina propria are defined as non-muscle-invasive bladder cancers (NMIBC), with intra-epithelial high-grade tumors classified as CIS (Tis). Tumors invading the muscularis propria are defined as muscle-invasive bladder cancer (MIBC) [5]. Complete transurethral resection (TUR), potentially followed by intravesical instillation of immunotherapeutic or chemotherapeutic agents, represents the ideal management strategy for NMIBC [6]. The standard treatment for patients with urothelial MIBC and MIBC with variant histologies is radical cystectomy, preceded in selected cN0M0 cases by neoadjuvant chemotherapy (NAC) [7]. Hematuria is the most common sign of bladder cancer. Papillary BC is eventually diagnosed by cystoscopic inspection of the bladder and histological assessment of collected tissue via cold-cup biopsy or resection. Cystoscopy and urine cytology can detect carcinoma in situ, and histological examination of bladder samples can confirm the diagnosis [8]. Cystoscopy is initially performed as an outpatient procedure and, despite being a relatively safe procedure, can cause discomfort, pain and infection and can be associated with high pre-procedural anxiety [9]. Conventional ultrasound (US), computed tomography (CT) urography and magnetic resonance imaging (MRI) are currently the most commonly used imaging modalities for assisting in the diagnosis and staging of bladder cancer [10]. Conventional US is a non-invasive, safe, affordable and very versatile imaging modality with a widespread use in different diseases and body areas. However, conventional US has limited use when it comes to certain anatomical structures and vascular lesions with low flow characteristics [11]. Contrast-enhanced ultrasound (CEUS) is a more recent US technique that can overcome some of the limitations of standard US via the intravenous administration of US contrast agents (UCAs) such as microbubbles [12]. In addition to its well-established role in echocardiography and liver lesions, CEUS is emerging as a diagnostic and staging tool in BC [13]. The technique is able to distinguish between benign tissue, such as clots or hematoma, and malignant lesions by perfusing the mass with contrast agent. Thanks to the different perfusion rates of the various layers of the bladder wall, CEUS is also able to differentiate between the mucosa, submucosa and muscle layer with high accuracy, thus predicting the tumor invasion depth and stage [14]. 

In this review, we analyze the imaging modalities currently used in BC diagnosing and staging and explain the CEUS technique and the rationale behind its use in BC patients. Using the Embase, PubMed and Web of Science research tool, we researched the available literature regarding the adoption of CEUS as a diagnostic and staging tool for BC and then described the results of the most influential studies in the field. 

The purpose of this review is to analyze the available literature regarding the use of CEUS in BC patients in order to further validate its use in BC diagnosis and staging, thus promoting a quick, economic and effective diagnostic tool for this high-impact disease.

## 2. Imaging Modalities for the Diagnosis and Staging of Bladder Cancer: Current Guidelines

European guidelines recommend the use of cystoscopy as standard of care for the initial diagnosis of bladder cancer. Cystoscopy can also be associated with hexaminolevulinate (HAL), an optical imaging agent used with blue light cystoscopy (BLC) in NMIBC diagnosis [15]. Increasing evidence from long-term follow-ups confirms the benefits of BLC over white light cystoscopy in terms of increased detection and reduced recurrence rates [15]. In addition to visual diagnosis, TURBT is still the standard method for histological diagnosis and for differentiating between NMIBC and MICBC.

Non-invasive techniques such as US provide intermediate sensitivity regarding a wide range of abnormalities in the upper and lower urinary tract, and it can be used as an addition to physical examinations. Although ultrasound cannot completely rule out all possible causes of hematuria, it does allow the imaging of intraluminal masses in the bladder, the characterization of renal masses and the detection of hydronephrosis [6]. 

Some of the difficulties associated with using traditional ultrasonographic testing to assess the bladder as a potential source of hematuria persist, despite notable advancements in diagnostic accuracy. Lesions that are tiny (less than 0.5 cm) and those that are situated in the bladder neck or dome are harder to see on conventional US. Another significant component is tumor confidence; plaque-like lesions are probably more difficult to find than polypoid ones [16]. Other limitations of traditional US that might lead to misdiagnosis are external factors, such as obesity of the patient and degree of bladder distension, while its adequacy depends on the experience and skill of the operator [17,18]. 

In order to overcome these limitations, more accurate methods have been developed in the diagnosis and staging of BC. More sophisticated radiologic imaging plays a major role in identifying local invasion, nodal status, distant metastasis and post-treatment surveillance.

In patients with hematuria, multidetector computed tomography urography (CTU) is helpful in identifying almost all cases of urothelial cancer. The detection of carcinoma in situ tumors with multidetector CTU is still a difficult task. Thus, the presence of CIS is not ruled out by negative results for urothelial carcinomas on CTU [19]. When compared to MRI (discussed later), CTU has a low sensitivity regarding differentiating NMIBC from MIBC and for local staging up to T3a. Nonetheless, it is one of the standard imaging techniques for nodal staging and distant metastasis [20].

Though CT has shown promise in detecting bladder tumors, multiparametric magnetic resonance imaging (mp-MRI) is being more extensively studied due to its better soft tissue imaging properties. In fact, it is not only able to identify solid or papillary tumors but can also predict the depth of bladder wall invasion with high accuracy [21]. A standardized methodology of MRI reporting (Vesical Imaging-Reporting and Data System [VI-RADS]) in patients with BC has recently been published and requires further validation [21].

To evaluate the bladder, VI-RADS combines dynamic contrast-enhancing (DCE), diffusion-weighted imaging (DWI) and T2-weighted (T2W) sequences. The low-intensity muscularis propria line, which can be used to assess muscle invasion, is mostly identified using T2W [21].

A first systematic review of eight studies showed that the VI-RADS scoring system can accurately differentiate NMIBC from MIBC with high inter-observer agreement rates [22].

MRI may be most promising in the pre-TURBT setting, given that the tissue architecture has not been distorted by surgery. Nonetheless, many tumors are understaged by TURBT or lack a detrusor sample; therefore, complementary imaging advancements are appreciated. On the other hand, MRI has low accuracy in the detection of carcinoma in situ.

The involvement of lymph nodes (LNs) in bladder cancer patients is a critical factor in determining their prognosis, and timely and effective therapeutic methods can only be identified by precise staging. To increase the accuracy of LN identification, 18F-FDG PET/CT has been used more frequently as a substitute for conventional techniques like CT or MRI, which are less accurate [23]. This method can provide important incremental staging and restaging information that can potentially influence clinical management, particularly in patients with muscle invasive bladder cancer.

Despite that, the need for nephrotoxic contrast agents, radiation exposure and the high cost and long duration of the exams are all limiting factors in the routine and widespread use of CTU, mp-MRI and 18F-FDG PET/CT for initial diagnosing and further staging of bladder lesions.

In this scenario, the evolution and standardization of an accurate, safe, quick and cost-effective method like CEUS could influence the development of future guidelines.

## 3. The Rationale behind the Use of CEUS in Bladder Cancer

The rationale behind the CEUS technique is based on infusion of a UCA in the bloodstream. The only known contraindications to UCAs are hypersensitivity to the substance and patent foramen ovale (PFO), but the latter contraindication might be rescinded in the future, as experts believe that existing evidence supports the judicious use of UCAs in PFO patients [24]. Different types of UCAs exist, such as microbubbles and nanodrops. The former is the most commonly used UCA type and is a gas/liquid emulsion with a stabilizing lipid or albumin (protein) shell [25]. Microbubbles filled with inert gas, such as sulfur hexafluoride (SHF), are preferred because they are more stable. SonoVue (Bracco SpA, Milan, Italy) is a second-generation UCA that comes with SHF and phospholipids [26]. Microbubbles have an average diameter of 3 μm [13], which is smaller than the wavelength of US, and this allows UCAs to circulate through blood capillaries until they reach the pulmonary circulation, where gas crosses the pulmonary filter [12]. Thanks to all these features, UCAs can reach microvascular networks surrounding and feeding a solid tumor, as in bladder cancer [27]. UCAs are thought to go through three phases once injected: the arterial phase (12–25 s after injection), the portal/venous phase (30–80 s after injection) and the late phase (>90 s after injection); a parenchymal phase is missing due to their features, and the enhancing effect lasts for about 8 min [12,13]. Adverse effects (AEs) of UCAs are rare and typically self-limiting. A 2019 single center study evaluated the safety of the SonoVue contrast agent in 34,478 patients. Only 0.12% of patients experienced AEs, and they were generally mild (mostly nausea, vomiting, dizziness and headache). Five cases required further treatment (three anaphylactic shock cases and two severe rashes) [28].

In the BC setting, CEUS is usually preceded by a B-mode US and a color Doppler ultrasound (CDUS). The bladder needs to be sufficiently filled, neither overfilled nor empty, in order to optimize bladder wall thickness for lesion detection [12]. Greyscale US has been adopted to characterize lesion morphology. According to its presentation, a lesion could be classified as sessile (lesion with a larger base compared to its height), polypoid (lesion with a taller height compared to its base), plaque-like (appearing as an elevation of the bladder wall without a discrete mass) or irregular (cauliflower-like) [12]. CDUS should be performed in order to evaluate the blood flow to lesions. In addition, micro-flow studies using power Doppler ultrasound (PDUS) may enhance evaluations of spatial resolution of blood flow in very small vessels by suppressing motion in surrounding tissues [29].

**Figure 1:**
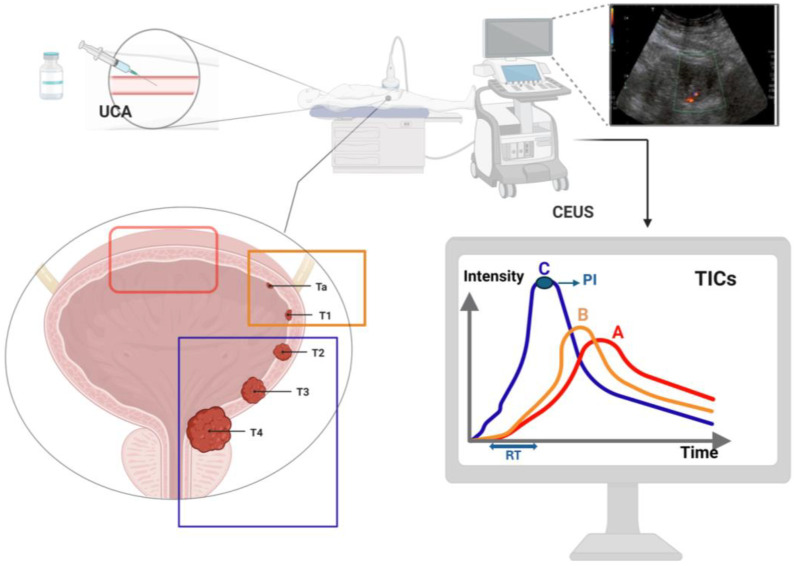
CEUS performance. Red box: Normal bladder wall; orange box: NMIBC; blue box: MIBC. UCA, ultrasound contrast agent; RT, rise time; PI, peak intensity. TICs (time intensity curves): curve A, normal bladder wall; curve B, low-grade carcinoma; curve C, high-grade carcinoma.

A CEUS examination starts with the infusion of UCA, i.e., 2.4 mL SonoVue, followed by a 5 mL flash of saline [12,13]. The examination should be performed using a lower-frequency convex probe, and the built-in contrast-specific CEUS imaging mode should be used. Once the probe is on the region of interest (ROI), the transducer has to be as still as possible. According to tissue enhancement with UCA, Drudi at al. developed a scoring system, with a score of 1 corresponding to a mild enhancement and a score of 2 referring to a strong enhancement. It is recommended to record the CEUS images to perform an offline analysis, in addition to a real-time evaluation. A minimum of two ROIs should be evaluated: one corresponding to the lesion and one in the adjacent normal bladder wall. Once the ROIs are constructed, specific software is used to calculate the contrast enhancement for every pixel for every second, thereby generating a time–intensity curve (TIC) and a parametric map (Figure 1). The TIC software allows one to visualize the rate of perfusion in the ROIs in a graphical format. Measured parameters usually include [12]:

- Wash-in time or rise time (RT), measured in seconds: the first point of signal intensity above the baseline;
- Peak intensity (PI), measured in dB: the maximum signal intensity in the selected ROIs;
- Time to peak (TTP), measured in seconds;
- Time from peak to one-half the signal intensity (TPH), measured in seconds: time from PI to one-half of PI;
- Mean transit time (MTT), measured in seconds: the time from the start of the rise of the TIC, past the PI and back to 50% of PI;
- Semi-descending slope (DS), measured in dB/second: the descending slope from maximum peak to half its intensity;
- Wash-out time: the length of time that precedes the moment at which the TIC returns to or nears zero after reaching the PI.

As already mentioned, neoangiogenesis is one of the hallmarks of tumor lesions. Tumor arterial vessels quickly lead to a rapid enhancement of the bladder mass, with high-grade tumors tending to show a rapid venous wash-out. Consequently, the CEUS technique leads to bladder lesion detection thanks to visualization of hyper-enhancing masses protruding into the bladder lumen or bladder wall thickening. Furthermore, CEUS allows for the exploration of the bladder wall in its layers in order to detect muscular invasion; upon UCA injection, the bladder wall can be seen separated into two enhancing layers: the lamina propria and the muscularis propria [12]. When the muscularis layer of the bladder is interrupted by the invading tumor, the lesion can be classified as an MIBC, while an NMIBC can be described when the relatively hypo-enhancing muscular layer of the bladder wall remains intact [30].

Another useful application of CEUS is that it enables one to differentiate between benign masses such as blood clots and malignant bladder lesions in an acute setting with a skilled radiologist where cystoscopy is not always feasible or would not provide adequate visualization due to excessive bleeding. Large blood clots that adhere to the bladder wall are especially challenging to differentiate from bladder cancer using conventional US, but when CEUS is adopted, the avascularity of the clots and lack of contrast enhancement makes them easily distinguishable. In a study by Liu et al., CEUS demonstrated better results in the differential diagnosis of intraluminal masses when compared to CDUS [31]. While CEUS can represent the real blood supply of a certain lesion, studies suggest that CDUS is not able to visualize vessels finer than 1–2 mm [32].

## 4. CEUS in Bladder Cancer: Review of the Available Literature

**Figure 2:**
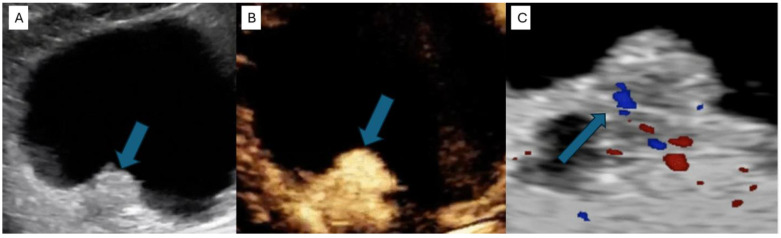
Comparison between greyscale US, CEUS and CDUS regarding the appearance of a solid tumor (indicated by a blue arrow). (**A**) On greyscale US, the tumor appears as an echogenic protuberance of the bladder wall. (**B**) The same mass shows a strong early enhancement after SonoVue injection, highly suggestive of malignancy. (**C**) CDUS shows vascularity mostly to the base of the lesion, raising suspicion for malignancy but less clearly than CEUS [34].

CEUS is able to identify the presence of a focal hyper-enhancing bladder wall thickening or an enhancing mass protruding into the bladder lumen (Figure 2). A 2014 study by Nicolau et al. showed a significant higher sensitivity and specificity of CEUS when compared to standard US in detecting bladder tumors. The accuracy of both approaches for detecting bladder cancer, as well as the number of discovered tumors, was analyzed and compared with the final diagnosis. The accuracy of baseline US in bladder cancer detection per patient was 72.09% (31/43 patients), with a sensitivity of 81.81% (27/33) and a specificity of 40% (4/10). By contrast, with a sensitivity of 90.9% (30/33) and a specificity of 80% (8/10), CEUS demonstrated a significantly higher accuracy of 88.37% (38/43 patients) [33].

A more recent series of 59 BC patients found that the accuracy of CEUS for detecting bladder lesions was 74.6%, which was slightly lower than MRI (76.3%) [35].

In addition to the demonstrated superiority of CEUS regarding its accuracy in detecting bladder cancer compared to conventional US, its main advantage is the possibility of predicting tumor grade and stage. A 2010 pilot study by Drudi et al. compared the specificities and sensitivities of CDUS and CEUS in the differentiation between low-grade and high-grade carcinoma using either the enhancement intensity score (1 = mild enhancement and 2 = strong enhancement) or by incorporating the shapes of the time intensity curves (TIC) (shape A = normal bladder, shape B = low-grade carcinoma and shape C = high-grade carcinoma) [36]. This study included 22 histologically confirmed high-grade carcinomas and 14 low-grade carcinomas. The sensitivity and specificity of CDUS were 86.4% and 42.9%, respectively. When using the enhancement intensity score for CEUS, the specificity and sensitivity increased to 90.9% and 85.7%, and to 95.4% and 85.7% when TIC was incorporated. Another study by Drudi et al. reported similar specificity and sensitivity, with all but eight (*n* = 56) low-grade carcinomas corresponding to TIC shape B and all but four (*n* = 88) high-grade carcinomas corresponding to TIC grade C [37]. In 2010, Caruso et al. evaluated the effectiveness of CEUS compared to conventional US in differentiating MIBC and NMIBC (defined as superficial bladder cancer at the time of the study). Using contrast-enhanced sonography, interruption of the muscle layer by enhancing tumor tissue was considered diagnostic of infiltration. A total of 34 patients were included. CEUS was able to identify all nine MIBCs, while conventional US only identified five of the nine muscle-infiltrating lesions. The diagnostic performance of CEUS reached that of the reference standard (AUC, 0.996), while the diagnostic accuracy of conventional US was worse (AUC, 0.613) [30].

A more recent study by Gupta et al. evaluated the role of CEUS in predicting both tumor grade and stage in 110 patients with BC who underwent CEUS prior to endoscopic resection [38]. The CEUS results were then compared with the final histology results. The final analysis revealed that CEUS was able to detect NMIBC with a sensitivity and specificity of 90% and 75.71% and MIBC with a sensitivity and specificity of 90.74% and 92.76%, respectively. Similar to Drudi et al., the authors used type B and type C TIC shapes to differentiate between low-grade (type B) and high-grade (type C) bladder cancer. CEUS predicted low-grade BC with a sensitivity, specificity, positive predictive value (PPV) and negative predictive value (NPV) of 78.12%, 85.14%, 69.44% and 90%, respectively. CEUS predicted high-grade BC with a sensitivity, specificity, PPV and NPV of 85.14%, 78.12%, 90% and 69.44%, respectively. In a 2021 systematic review and meta-analysis, Ge et al. evaluated the accuracy of CEUS in differentiating between stage Ta-T1, or low-grade BC, and stage T2, or high-grade BC [39]. Five studies (see Table 1) met the selection criteria, with a total of 436 BC patients. The pooled sensitivity (P-SEN) and pooled specificity (P-SPE) were 94.0% (95% CI: 85–98%) and 90% (95% CI: 83–95%), respectively, which is similar to those reported for MRI in a recent systematic review [40]. The pooled positive likelihood ratio (PLR+) was 9.5 (95% CI: 5.13–17.6), and the pooled negative likelihood ratio (PLR−) was 0.06 (95% CI: 0.02–0.17), demonstrating strong diagnostic accuracy. In another recent study by Fu et al., 160 BC patients underwent both contrast-enhanced MRI and CEUS for the diagnosis and staging of bladder lesions (Table 1). The results confirmed superimposable accuracies of the two imaging modalities for the detection of BC (85.90% for CEUS and 84.62% for MRI, *p* > 0.05). In addition, CEUS was found to have a better sensitivity in detecting muscularis invasion, with accuracies of 84.72% for CEUS and 76.92% for MRI [41].

## 5. Conclusions

CEUS is a highly accurate diagnostic and staging tool for BC, reaching levels of specificity and sensitivity in differentiating between Ta-T1, or low-grade BC, and T2, or high-grade BC, comparable to those shown by the reference standard methods. In addition to its accuracy, it is also a more affordable, safe and versatile imaging modality compared to MRI or CT [44]. UCAs do not leak into the extravascular space, and, in contrast with MRI and CT contrast agents, they can be safely used in patients with reduced GFR or liver dysfunction and do not show any long-lasting adverse effects [45]. This is particularly useful in the BC setting, as it is common to encounter patients with elevated serum creatinine levels due to obstructive uropathy who cannot undergo MRI or CT urography because of the risk of nephropathy or nephrogenic systemic fibrosis [46].

The main limitation of CEUS is the difficulty of detecting flat or plaque-like lesions and tumors that are <5 mm in diameter due to a lack of significant neoangiogenesis and thus the absence of increased enhancement. In a study by Nicolau et al., with tumors < 5 mm, CEUS demonstrated a sensitivity of 20%, but when only lesions > 5 mm were considered, the sensitivity rose to 94.9% [33]. In addition, while MRI and CT are more invasive imaging techniques, they have the advantage of providing additional information regarding nodal (MRI and CT) and systemic (CT) staging that cannot be acquired through CEUS. Moreover, the use of UCAs cannot overcome some of the main limiting factors of conventional ultrasound, such as obesity, meteorism and calcifications, as well as the difficulty of assessing lesions that are located in the bladder dome or floor [47]. Another limitation of conventional US that CEUS cannot overcome is a dependence on the operator’s level of experience, with even longer learning curves than conventional ultrasound [48,49].

In conclusion, in spite of its limitations, CEUS has proved to be a safe and effective method for the detection and staging of bladder cancer, and, if adopted in the correct setting, it will likely have results that are similar to the diagnostic tools currently recommended [50]. Despite that, the present study is merely a descriptive review, and further validation through randomized controlled trials and meta-analyses is needed before CEUS can be included in BC guidelines.

## Figures and Tables

**Table 1:** Available studies evaluating the role of CEUS in bladder cancer.

Author, Year	Country	Study Design	Patients, N	Male (%)	Reference Standard	Sen	Spe	Aim
Caruso, 2010 [30]	Italy	Prospective	34	94.1	Cystoscopy and TURBT	1	0.913	Discrimination NMIBC vs. MIBC
Drudi, 2012 [37]	Italy	Prospective	144	66.6	Cystoscopy and TURBT	0.909	0.857	Discrimination High-grade vs. Low-grade
Li, 2012 [42]	China	Prospective	60	75	Cystoscopy and TURBT	1	0.857	Discrimination NMIBC vs. MIBC
Gupta, 2016 [38]	India	Prospective	110	87.3	TURBT	0.90	0.75 vs. 0.928	Discrimination NMIBC vs. MIBC
Li, 2017 [43]	China	Retrospective Prospective	96 96	NR	TURBT	0.857	0.850	Discrimination High-grade vs. Low-grade
Fu, 2022 [41]	China	Retrospective	160	66.2	TURBT	0.853	0.833	Discrimination NMIBC vs. MIBC
Baoming Luo, Ongoing (NCT05204108)	China	Prospective	NR	NR	TURBT	NR	NR	Discrimination NMIBC vs. MIBC

TURBT: Transuretral resection of bladder tumour; NR: Not reported.
